# Transcriptomic and Phenotypic Analysis of a *spoIIE* Mutant in *Clostridium beijerinckii*

**DOI:** 10.3389/fmicb.2020.556064

**Published:** 2020-09-15

**Authors:** Mamou Diallo, Nicolas Kint, Marc Monot, Florent Collas, Isabelle Martin-Verstraete, John van der Oost, Servé W. M. Kengen, Ana M. López-Contreras

**Affiliations:** ^1^Wageningen Food and Biobased Research, Wageningen, Netherlands; ^2^Laboratory of Microbiology, Wageningen University, Wageningen, Netherlands; ^3^Laboratoire Pathogènese des Bactéries Anaérobies, Institut Pasteur, UMR CNRS 2001, Université de Paris, Paris, France; ^4^Biomics platform, C2RT, Institut Pasteur, Paris, France; ^5^Institut Universitaire de France, Paris, France

**Keywords:** *Clostridium beijerinckii* NCIMB 8052, sporulation, spoIIE, ABE production, CRISPR-Cas9, RNA seq, transcriptome analysis

## Abstract

SpoIIE is a phosphatase involved in the activation of the first sigma factor of the forespore, σ^*F*^, during sporulation. A Δ*spoIIE* mutant of *Clostridium beijerinckii* NCIMB 8052, previously generated by CRISPR-Cas9, did not sporulate but still produced granulose and solvents. Microscopy analysis also showed that the cells of the Δ*spoIIE* mutant are elongated with the presence of multiple septa. This observation suggests that in *C. beijerinckii*, SpoIIE is necessary for the completion of the sporulation process, as seen in *Bacillus* and *Clostridium acetobutylicum*. Moreover, when grown in reactors, the *spoIIE* mutant produced higher levels of solvents than the wild type strain. The impact of the *spoIIE* inactivation on gene transcription was assessed by comparative transcriptome analysis at three time points (4 h, 11 h and 23 h). Approximately 5% of the genes were differentially expressed in the mutant compared to the wild type strain at all time points. Out of those only 12% were known sporulation genes. As expected, the genes belonging to the regulon of the sporulation specific transcription factors (σ^*F*^, σ^*E*^, σ^*G*^, σ^*K*^) were strongly down-regulated in the mutant. Inactivation of *spoIIE* also caused differential expression of genes involved in various cell processes at each time point. Moreover, at 23 h, genes involved in butanol formation and tolerance, as well as in cell motility, were up-regulated in the mutant. In contrast, several genes involved in cell wall composition, oxidative stress and amino acid transport were down-regulated. These results indicate an intricate interdependence of sporulation and stationary phase cellular events in *C. beijerinckii*.

## Introduction

Even though butanol is nowadays mainly produced through the petrochemical route, it used to be made industrially by a bioprocess called ABE fermentation in the first half of the 20th century. This process returned to the forefront at the end of the 1990s with the emerging interest for biobased chemicals. ABE fermentation relies on the ability of several bacteria from the *Clostridium* genus to convert carbohydrates to acetone, ethanol, butanol (ABE) and isopropanol. Clostridia are anaerobic bacteria that can form spores to protect themselves from unfavorable environmental conditions, including oxygen exposure. The main representatives of the solventogenic clostridia group are *Clostridium acetobutylicum*, *C. beijerinckii*, *C. saccharobutylicum* and *C. saccharoperbutylacetonicum*. These clostridia produce solvents while they form spores. Once the spores are mature, the solvent producing cells lyse, and the metabolically inactive spores are left behind (Al-Hinai et al., 2015). That is why in industry, spores are seen as undesirable (Tracy et al., 2012; Li et al., 2020), and many efforts were made to engineer asporogenous solvent producing strains (Scotcher and Bennett, 2005; Bi et al., 2011; Al-Hinai et al., 2014). However, the sporulation process and the associated regulatory network in these microorganisms are still poorly characterized (Patakova et al., 2013).

In solventogenic clostridia, several studies revealed a link between sporulation and solvent production (Al-Hinai et al., 2014, 2015) but, none was able to explain the involved mechanism. The regulatory pathway controlling sporulation was first described and intensively studied in *Bacillus subtilis*, which is considered as a model organism for the sporulation process. Comparative studies between bacilli and clostridia show similarities in the sporulation process and its regulation, including the presence of the main actors such as Spo0A and σ^*H*^ as well as the four sporulation specific sigma factors, σ^*F*^, σ^*E*^, σ^*G*^ and σ^*K*^ (Al-Hinai et al., 2015). However, differences in the sporulation regulatory networks are also observed between bacilli and clostridia and even among clostridia (Al-Hinai et al., 2015). Important deviations from the *B. subtilis* paradigm exist in clostridial spore formers, especially concerning the communication between the forespore and the mother cell, a weaker connection between gene expression and morphogenesis, and modifications in the interplay between sigma factors (Paredes et al., 2005; Fimlaid and Shen, 2015).

**FIGURE 1:**
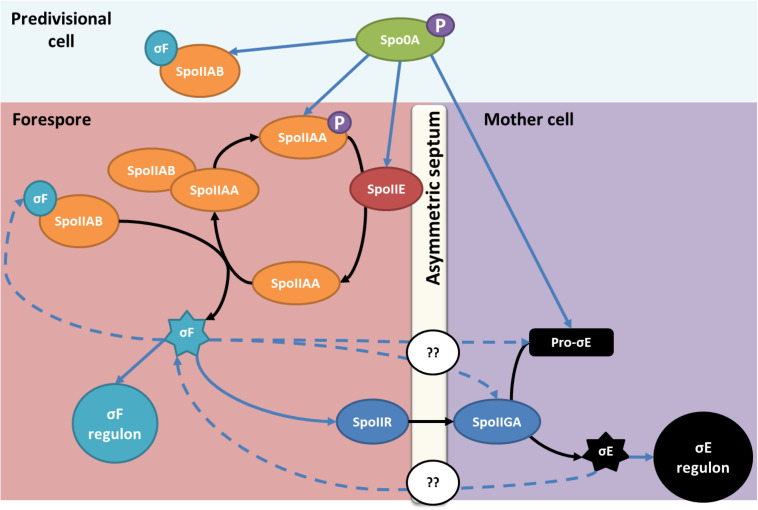
SpoIIE, an essential protein for sporulation in *B. subtilis* and *C. acetobutylicum* in stage II of sporulation. After sporulation initiation, the expression of *spoIIE* and the *spoIIAA-spoIIAB-sigF* operon is promoted by phosphorylated Spo0A. In *B. subtilis* and *C. acetobutylicum*, SpoIIE dephosphorylates SpoIIAA, the anti-anti sigma factor that sequestrates σ^*F*^. The dephosphorylated SpoIIAA binds to SpoIIAB and enables the release of σ^*F*^ in the prespore. The blue arrows indicate an impact on the gene transcription; dotted arrows are only valid for *C. acetobutylicum.* In contrast to what is observed in *Bacillus*, σ^*F*^ and σ^*E*^ impact events in both the prespore and the mother cell compartment in *Clostridium*, however, the mechanism enabling the interaction is unknown. Adapted from Al-Hinai et al. (2015) and amended with permission from American Society for Microbiology.

In *B. subtilis*, the SpoIIE protein is a phosphatase that plays a crucial role in the sporulation regulation mechanism (Barák et al., 2019). SpoIIE acts in stage II of the sporulation process. SpoIIE plays a central role in the asymmetric septum formation separating the mother cell and the forespore. Studies in *B. subtilis* showed that SpoIIE interacts with cell division proteins and peptidoglycan synthesis proteins to enable a correct localization and thickness of the asymmetric septum (Feucht et al., 2002; Muchová et al., 2016; Muchová et al., 2020). Following the asymmetric division, SpoIIE enables the activation of σ^*F*^, the first sigma factor of the forespore (Figure 1; Louie et al., 1992; Levin and Losick, 1994). σ^*F*^ is held inactive by the anti-sigma factor and kinase SpoIIAB. At the beginning of stage II of the sporulation cascade, SpoIIE dephosphorylates the anti-anti-sigma factor SpoIIAA to enable its interaction with SpoIIAB, which releases σ^*F*^ following asymmetric division (Al-Hinai et al., 2015; Figure 1). In *C. acetobutylicum*, considered as the model solventogenic *Clostridium*, SpoIIE has also been reported to function as a phosphatase involved in the early stages of the sporulation regulation cascade. Previous studies have also shown that *spoIIE* mutants of *C. acetobutylicum* were asporogenous but still produced solvents (Scotcher and Bennett, 2005; Bi et al., 2011). However, no studies to date confirmed if this model can be applied to other ABE-producing strains.

*Clostridium beijerinckii* is the second most studied solventogenic specie and was used industrially for acetone production already at the beginning of the 20th century (Jones and Woods, 1986). *C. beijerinckii* is known for its ability to catabolize a wide range of carbohydrates (van der Wal et al., 2013; Gu et al., 2014). However, few studies on the sporulation mechanism have been conducted in this species. We have recently inactivated the *spoIIE* gene (Cbei_0097) in *C. beijerinckii* NCIMB 8052 using a CRISPR-Cas9 system for Clostridia developed in our laboratory (Diallo et al., 2020). In this work, we analyzed the impact of *spoIIE* inactivation in *C. beijerinckii* NCIMB 8052 on the sporulation process and the solvent production through fermentation assays, microscopy observations and transcriptome analysis.

## Materials and Methods

### Bacterial Strains and Culture Conditions

Bacterial strains and plasmids are listed in Table 1. The *C. beijerinckii* wild-type (WT) strain was stored as spore suspension and the mutants as vegetative cells in 15% glycerol solution at – 20°C. Spore suspensions were heat-shocked 1 min at 98°C before inoculation in a liquid medium to kill any vegetative cell present and enable the germination of the spores. Except for fermentation assays, liquid cultures of the WT, mutant and complemented strains were grown in liquid modified CGM (mCGM) as described previously (Kuit et al., 2012) containing per liter: yeast extract, 5 g; KH_2_PO_4_, 0.75 g; K_2_HPO_4_, 0.75 g; asparagine⋅H_2_O, 2 g; (NH_4_)_2_SO_4_, 2 g; cysteine, 0.50 g; ⋅MgSO_4_7⋅ H_2_O, 0.40 g; MnSO_4_⋅H_2_O, 0.01 g; FeSO_4_7⋅ H_2_O, 0.01 g; glucose, 10 g. Liquid media were made anaerobic by flushing with nitrogen gas. Cultivation was performed at 35°C anaerobically in an anaerobic chamber without shaking (Sheldon Manufacturing, United States). The gas mixture used consists of 15% CO_2_, 4% H_2_ and 81% N_2_. *Clostridium* strains were grown and selected on modified CGM agar described previously (de Gérando et al., 2016) containing per liter: yeast extract, 1 g; tryptone, 2 g; KH_2_PO_4_, 0.50 g; K_2_HPO_4_, 1 g; (NH_4_)_2_SO_4_, 2 g, MgSO_4_7⋅ H_2_O, 0.10 g; MnSO_4_H_2_O, 0.01 g; FeSO_4_⋅7 H_2_O, 0.015 g; CaCl_2_, 0.01 g; CoCl_2_, 0.002 g; CaCl_2_, 0.002 g; glucose, 50 g and agar 12 g. *Escherichia coli* XL1 blue cells (Agilent, United States) were used for cloning. *E. coli* strains were stored at −80°C in the presence of 15% glycerol. *E. coli* was grown on LB medium, supplemented with 100 μg/mL spectinomycin (Duchefa, Netherlands).

**TABLE 1:** Strains and plasmids used in this study; *catP*, chloramphenicol resistance gene; *aad9*, spectinomycin resistance gene.

Strains or plasmids	Relevant characteristics	Reference
**Strains**		
*C. beijerinckii* NCIMB 8052 (WT)	Wild type, sensitive to spectinomycin (650 μg/mL) and erythromycin (25–50 μg/mL)	NCIMB
*C.beijerinckii* Δ*spoIIE*	NCIMB 8052, Δ*spoIIE*	(Diallo et al., 2020)
*E. coli* XL1-blue	recA1 endA1 gyrA96 thi-1 hsdR17 supE44 relA1 lac [F′ proAB lacIq ZΔM15 Tn10 (Tetr)]	Agilent
**Plasmids**		
pSpoIIE	pCB102, colE1, aad9, Cbei_0097	(Diallo et al., 2020)
pRAN473	repA, colE1, Ptet:mCherryOpt–MCS, catP	(Ransom et al., 2015)
pRAN473S	repA, colE1, Ptet:mCherryOpt–MCS, aad9	This study
pRAN473S: cbei_0097	repA, colE1, Ptet:mCherryOpt–cbei_0097, aad9	This study

### Plasmid Construction

The primers used for plasmid construction are listed in the Supplementary Table 1 and were synthesized by Integrated DNA Technologies. To replace the *catp* gene by *aad9* in pRAN473, pRAN473 was linearized by PCR using the primers M392 and M393. The *aad9* gene was amplified from pSpoIIE with the primers M394 and M395. The fragments were fused using the Circular Polymerase Extension Cloning (Quan and Tian, 2014) to generate pRAN473S. pRAN473S:cbei_0097 was constructed according to the protocol from Ransom et al. (2016). The *spoIIE* gene (Cbei_0097) was amplified from pSpoIIE by PCR using M396 and M397. The obtained PCR product and pRAN473S were digested by Sal1 and BamH1. The fragments were ligated using the T4 DNA ligase (New England Biolabs, United States) according to the manufacturer protocol.

### Fermentation

Fermentations were performed at 35°C in CM2 medium (van der Wal et al., 2013), which contains per liter: yeast extract, 5 g; KH_2_PO_4_, 1 g; K_2_HPO_4_, 0.76 g; ammonium acetate, 3 g; *p*-aminobenzoic acid, 0.10 g; MgSO_4_⋅7 H_2_O, 1 g; and FeSO_4_⋅7 H_2_O, 0.50 g, glucose, 60 g. Metabolites were determined in culture supernatants after removal of cells by centrifugation. Glucose, acetate, butyrate, lactate, acetone, butanol and ethanol concentration in the culture medium were determined by high-performance liquid chromatography (HPLC) as described previously using 4 methyl valeric acid (30 mM) as an internal standard (Collas et al., 2012; Kuit et al., 2012).

### DNA Extraction and Sequencing

Genomic DNA of *C. beijerinckii* NCIMB 8052 and *C. beijerinckii* Δ*spoIIE* mutants was purified using the GenElute bacterial genomic DNA kit (Sigma-Aldrich, United States). The concentration of genomic DNA was determined using a nanodrop spectrophotometer (Thermo Fisher Scientific, United States) and quality checked on 1% agarose gel. PCR reactions were carried out using the Q5 Master mix (New England Biolabs, United States). DNA sequencing of clones and genome assembly were performed by BaseClear (Leiden, Netherlands). The sequences of the WT and the Δ*spoIIE* clones were compared to the publicly available sequence of *C. beijerinckii* NCIMB 8052 on NCBI. SNPs between the genome of our WT and the Δ*spoIIE* mutant’s genome with a frequency above 98% were considered in our study.

### Granulose Staining

Granulose accumulation was monitored by iodine staining. Each *C. beijerinckii* strain was grown on CM2 agar plates and incubated anaerobically at 37°C. After 24 h of incubation, the petri dish was opened and inverted over I_2_ crystals for approximately 1 min. The colonies of granulose-negative mutants were unstained by the sublimed I_2_ vapors, while the granulose-positive strains were labeled (Robson et al., 1974; Steiner et al., 2012).

### Spore Viability Assay

To verify the presence of viable spores, overnight cultures of each strain were prepared in 5 mL CM2 medium. The next day, two tubes containing 15 mL of fresh CM2 liquid medium were inoculated with 50 μL of overnight culture. Aliquots of 100 μL were collected at 24 h and 48 h, treated at 98^*o*^C for 1 min and used for 4 serial dilutions. 50 μL of each dilution was spread on CM2 agar plates in the anaerobic chamber and incubated for 24 h at 37°C. The colonies on each plate were then counted to determine the average number of viable spores per mL (spores/mL). This method was adapted from (Steiner et al., 2011).

### Microscopy Analysis

Phase-contrast microscopy (Olympus BX51) was used to observe the morphology of WT and Δ*spoIIE* strains at ×400 and x1000 magnifications. Cells were cultivated for 72 h in liquid CM2 medium, samples were collected at 48 and 72 h, and centrifuged to stain them according to the Schaeffer-Fulton technique. A cell film was made on a glass slide and stained with malachite green and safranin to visualize spores (Schaeffer and Fulton, 1933; Al-Hinai et al., 2014). The stained cells were observed by phase-contrast microscopy at x1000 magnification to detect the presence of spores. For the fluorescent microscopy analysis, cells were washed three times by centrifugation (5000 g, 3 min) and resuspended in 1 mL of PBS. Following washing, the cells were resuspended in 1 mL of PBS supplemented with the membrane dye Mitotracker Green (MTG, 0.5 mg.mL^–1^) (Molecular Probes, Invitrogen Thermo Fisher Scientific, United States). Cells were mounted on 1.7% agarose coated glass slides and observed in a home built and designed microscope. Fluorescent signals were visualized with a Nikon CFI SR HP Apochromat TIRF 100XC Oil objective, and images were captured using an Andor Zyla 4.2 PLUS sCMOS camera. To observe the localization of the SpoIIE protein, the *spoIIE* gene was fused to a mCherry coding sequence, and the translational fusion was expressed under the control of the Ptet promoter inducible in the presence of anhydrotetracycline (atc). *C. beijerinckii* Δ*spoIIE* cells harboring the pRAN473S and pRAN473S:cbei_0097 were grown in CM2 containing 650 μg/mL of spectinomycin. After 10 h of growth, atc (Sigma-Aldrich, United States) was added at 200 ng/mL. Samples for microscopy analysis were collected 8 h after induction. Before MTG staining, the cells were fixed as previously described (Ransom et al., 2016).

### Transcriptome Analysis

#### RNA Isolation and Sequencing Protocol

The same procedure was repeated three times in three different weeks to obtain three independent biological replicates. Each week, a fresh preculture was used to inoculate two identical bioreactors. The cultures were grown, as described in section “Fermentation.” Samples were taken over the early exponential, late exponential and stationary phases (samples at 4, 11 and 23 h). Following centrifugation of the samples, cell pellets were washed with chilled RNase free water and resuspended in RNase free water to obtain a suspension having an OD_600__*nm*_ of approximately 1. A 3-mL diluted sample was centrifuged, the supernatant was discarded, and the cell pellet was stored at −80°C for subsequent isolation. Frozen samples were thawed on ice, and RNA was isolated using High pure RNA isolation kit (Roche Diagnostics, Switzerland). Quality and concentration of RNA samples were checked using a nanodrop spectrophotometer (Thermo Fisher Scientific, United States). The absence of DNA in the RNA samples was evaluated by qPCR analysis performed with BioRad CFX 96 Touch^TM^ (BioRad, Hercules, United States) and the PowerUP SYBr green reaction mix (Applied Biosystems, Thermo Fisher Scientific, United States). Reactions were performed in an overall volume of 10 μL with concentrations of components and reaction conditions, as described in the master mix protocol. RNA quality and integrity were determined using the Qsep 100 bioanalyzer (Bioptic Inc., Taiwan).

All the RNA samples collected were used for library construction and sequencing. The Ribo-zero kit (Illumina, United States) was used to enrich the samples in mRNA. The stranded library was prepared using the TruSeq Stranded mRNA Library Prep Kit (Illumina, United States) according to the manufacturer’s recommendation. AMPure XP beads (Beckman Coulter, United States) were used to clean up the cDNA fragments after each process. Library quality was checked using the bioanalyzer, and the library was then loaded onto HiSeq 2500 for high-throughput sequencing.

### Bioinformatics Analysis

After Illumina sequencing, all the reads were mapped to the *C. beijerinckii* genome using Bowtie (Langmead et al., 2009) then converted into BAM files with the Samtools (Li et al., 2009). Differential analysis of RNA-seq data and statistical analysis were performed with Sartools pipeline (Varet et al., 2016) using the DESeq2 package. The data were visualized in a strand-specific manner using COV2HTML^1^ (Monot et al., 2014). Genes were considered differentially expressed when padj < 0.05 and gene expression with a | log_2_ fold change | > 1.5. Gene expression was considered down-regulated if log_2_ fold change < −1.5 or up-regulated if log_2_ fold change > 1.5.

RT-qPCR analyses were performed with BioRad CFX 96 Touch^TM^ and the PowerUP SYBr green reaction mix to confirm the RNA seq results. Primer3 website was used for oligonucleotide design (Supplementary Table S2). Relative expression at 23 h of 12 genes was monitored (Supplementary Table S2); gene *gyrA* was chosen as the reference gene from a selection of candidate genes (including *gyrA, 16S rna, polIII* and *alaS*) based on analysis by RefFinder algorithms to verify the stability of their expression. Reaction efficiency was determined for each assay using a ×5 serial dilution of cDNA samples. A sample collected after 4 h of cultivation in CM2 medium was chosen as a calibrator and a reference value for calculation of expression fold-change of each gene in other samples. All RT-qPCR analyses were performed in triplicate. The relative quantification was evaluated using the mathematical model described by Dr. Pfaffl (Pfaffl, 2001).

## Results

### SpoIIE Disruption and Mutant Characterization During the Sporulation Cycle

**FIGURE 2:**
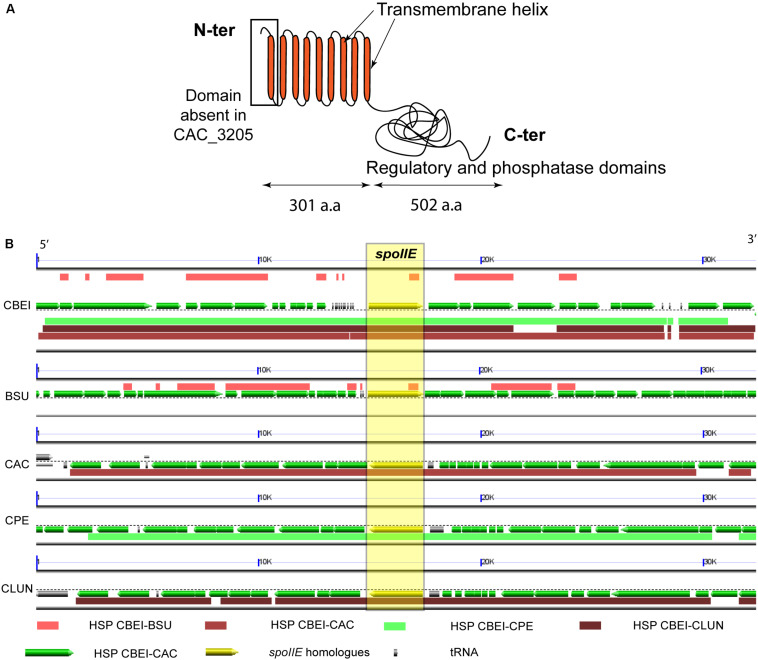
SpoIIE a sporulation protein conserved in spore-forming Firmicutes **(A)** Illustration of the predicted SpoIIE protein with its transmembrane regions (according to the TMHMM software) and its phosphatase domain terminus, a.a: amino acids **(B)** Synteny map of the *spoIIE* region from Cbei_0084 to Cbei_0107, with *spoIIE* (Cbei_0097), in *C. beijerinckii* NCIMB 8052 (CBEI) and its homologs in *B. subtilis* str.168 (BSU), *C. acetobutylicum* ATCC 824 (CAC), *C. perfringens* str.13 (CPE) and *C. ljundahlii* ATCC 49587 (CLUN), the homologous regions (HSP) are highlighted by color blocks for each organism. Image generated using the COGE platform https://genomevolution.org/r/1ceci, and the GEvo tool (Tang et al., 2015).

The *spoIIE* gene (Cbei_0097) from *C. beijerinckii* NCIMB 8052 is 2412 bp long and encodes an 803 amino acid long transmembrane protein. The *spoIIE* gene is present in all the strains of *C. beijerinckii* with a genome available. In all *C. beijerinckii* strains, *spoIIE* shows a very high level of identity ranging from 96% to 100%. SpoIIE orthologs were found in all spore-forming Bacilli and Clostridia (Galperin et al., 2012). In *Clostridium*, its role was studied only in *C. acetobutylicum* ATCC 824, where SpoIIE (Cac_3205, 795 amino acids) was reported to be involved in sporulation, but not to affect directly solvent production (Scotcher and Bennett, 2005; Bi et al., 2011). SpoIIE of *C. beijerinckii* NCIMB 8052 (cbei_spoIIE) shows 36% identity [62% similarity] with SpoIIE of *C. acetobutylicum* ATCC 824, 26% identity with SpoIIE of *B. subtilis* and 42% identity with SpoIIE of *Clostridium perfringens*. However, compared with SpoIIE of *C. acetobutylicum*, the protein of *C. beijerinckii* is longer. An extra transmembrane helix at the N terminus of the *C. beijerinckii* protein (Figure 2A) was predicted by the TMHMM software (Krogh et al., 2001). This region is conserved in *B. subtilis* and *C. perfringens*. Synteny of the genomic region, in which the *spoIIE* gene is located, was observed in *Clostridium* and *Bacillus* (Figure 2B). Indeed in *C. beijerinckii* NCIMB 8052 and *C. acetobutylicum* ATCC 824, *spoIIE* is in the middle of a conserved genomic region larger than 30 kb harboring more than 20 genes (Figure 2B). Moreover, SpoIIE inactivation in *B. subtilis* and *C. acetobutylicum* resulted in an asporogenous phenotype (Barák and Youngman, 1996; Scotcher and Bennett, 2005; Grage et al., 2017), and the bacteria could not complete stage II of the sporulation cycle (Al-Hinai et al., 2015). All these observations suggest that the protein has a similar function in *Bacillus* and *Clostridium*.

**FIGURE 3:**
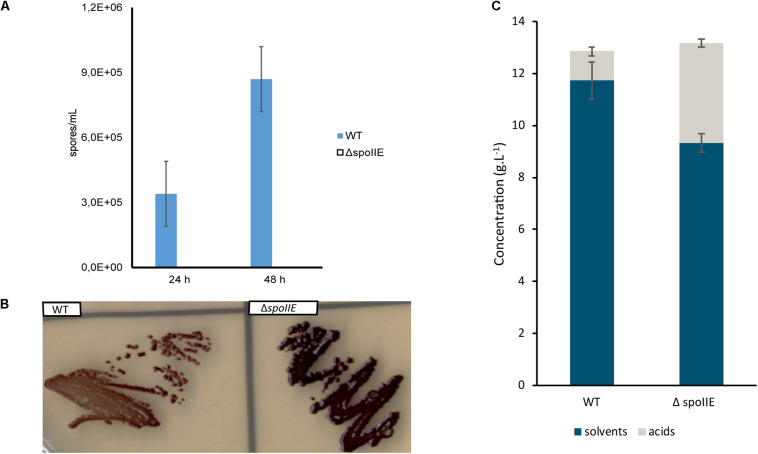
Phenotypic comparison of Δ*spoIIE* mutant and WT. The Δ*spoIIE* mutant could not produce viable spores but still produced granulose and solvents. **(A)** Spore viability assay; **(B)** Granulose detection after 24 h of incubation; **(C)** Fermentation end products after 72 h of culture at 35°C in 50-mL serum bottles, the error bars indicate one standard deviation of the mean, which was determined based on the data from biological duplicates (*n* = 2).

Using a xylose inducible CRISPR-Cas9 system, we recently constructed a *spoIIE* mutant by deleting a 2.379 kb fragment of the coding sequence of *spoIIE* (Diallo et al., 2020). The genomes of four independent mutants were sequenced to confirm the *spoIIE* deletion and the absence of any other mutations. Compared to the genome of the WT strain used in our laboratory, the mutants’ genomes harbored between 7 and 23 SNPs (Supplementary Table S3). The C5 mutant harbored the fewest modifications. In this mutant, only 7 SNPs were detected. Out of these SNPs, 4 were located in CDS, of which 3 were found in the genome of the other mutants. The mutant C5 was selected for further studies of the *spoIIE* mutant. After 24 h and 48 h of growth in liquid medium, samples of C5 and WT cultures were collected and incubated at 98°C for 1 min and then plated on solid medium to determine their sporulation efficiency. This heat treatment kills the vegetative cells and induces spore germination. No viable spores were detected in the C5 culture, as no colonies grew on the respective plates, in contrast to the growth observed in the plates corresponding to the WT cultures (Figure 3A). Sporulation capacity was partially restored once the C5 mutant was complemented (Supplementary Figure S1) with a plasmid carrying a functional *spoIIE* gene with its native promoter (Diallo et al., 2020), confirming the direct link between *spoIIE*’s disruption and the mutant’s asporogenous phenotype. After incubation of the strains for 24 h on plates and iodine staining, we observed that both the mutant and WT strains produced granulose (Figure 3B). Moreover, when grown in serum bottles in CM2 medium, the Δ*spoIIE* mutant metabolized glucose and produced solvents even if the global amount of solvent was reduced and the production of acid increased as compared to the WT strain (Figure 3C). SpoIIE inactivation abolished sporulation but did not prevent granulose nor solvent production.

**FIGURE 4:**
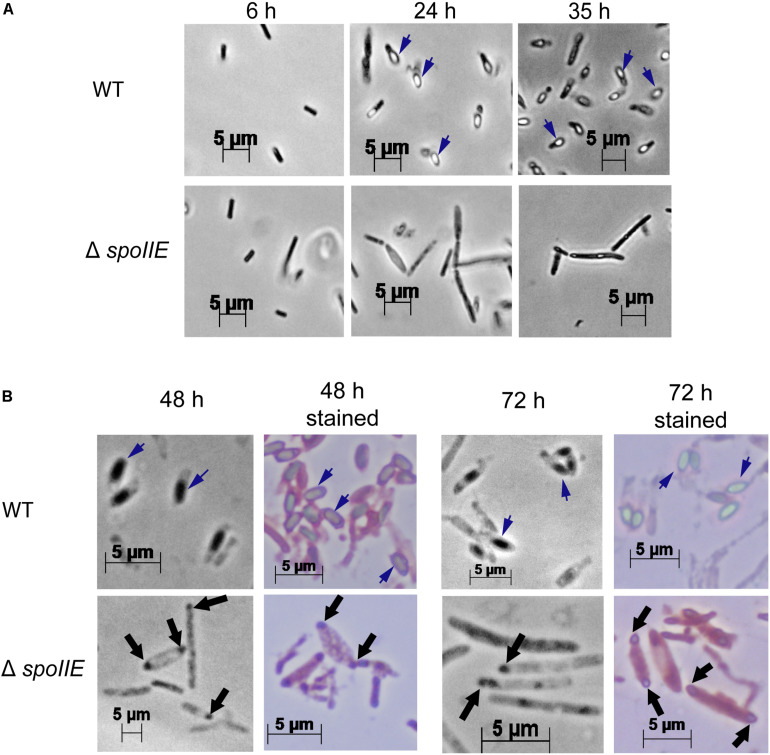
Phase-contrast microscopy images of *C. beijerinckii* WT and Δ*spoIIE* cells during the fermentation. While mature spores were seen in the WT images after 24 h of cultivation, no spores were seen in Δ*spoIIE* cultures even after 72 h of cultivation. However phase-dark bodies, stained in blue by Shaeffer-Fulton staining, were observed in the Δ*spoIIE* cells after 48 h of cultivation **(A)** Pictures with x400 magnification at 6 h, 24 h and 35 h; **(B)** Pictures at 48 h and 72 h of culture with x1000 magnification with and without Shaeffer-Fulton stain. The short dark blue arrows indicate mature spores. The black arrows indicate the phase-dark masses at the poles of the mutant cells.

**FIGURE 5:**
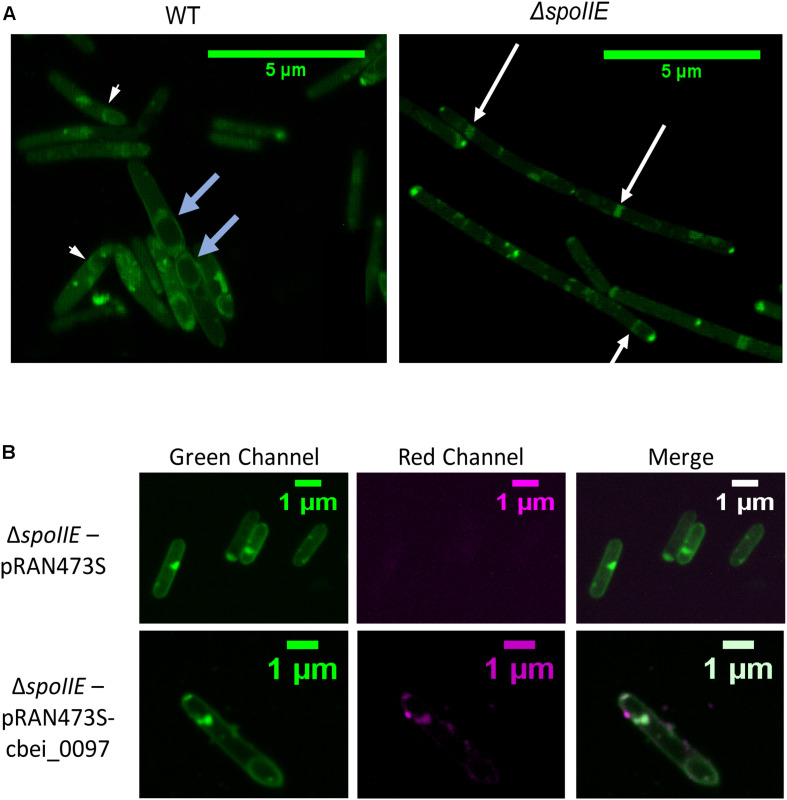
Fluorescence microscopy images of *C. beijerinckii* WT and Δ*spoIIE* cells **(A)** Fluorescence images of the wild type and the mutant cells after 20 h of cultivation and stained by the membrane staining MTG; **(B)** Fluorescence images of the Δ*spoIIE* mutant cells stained by MTG and harboring either the mCherry empty plasmid (pRAN73S) or the plasmid expressing mCherry fused to Cbei_0097 (pRAN73S:cbei_0097) at 18 h of cultivation (after 8 h of atc induction). The white arrows indicate septa observed in the mutant strain. The short white blue arrows indicate wild type cells with an asymmetric septum. The light blue arrows indicate the prespores observed in the wild type.

We observed the morphology of the cells after 6 h, 24 h, 35 h, 48 h and 72 h of cultivation with phase-contrast microscopy (Figure 4) and after 20 h with fluorescent microscopy (Figure 5A, Supplementary Figures S2A,B). While mature spores were detected after 24 h of growth in WT cultures, no regular pre-spores nor spores were seen in Δ*spoIIE* mutant cultures, even after 72 h of incubation (Figure 4). At 48 h and 72 h, several mutant cells harbored phase-dark masses at the poles of the cells (Figure 4B black arrows) that were absent in WT cells. Even in the complemented Δ*spoIIE* mutant, in which sporulation was restored, some cells still harbored these phase-dark bodies at the poles (Diallo et al., 2020). These masses were stained by malachite green (Figure 4B) and could be a result of DNA condensing in forespore like compartments, as observed in the *sigE* mutant of *C. acetobutylicum* (Tracy et al., 2011) and *spoIIE* mutants of *B.subtilis* (Barák and Youngman, 1996). Moreover, mutant cells had a different morphology compared to WT cells, from 20 h of cultivation onward, as observed by fluorescent microscopy after MTG labeling. The cells of the Δ*spoIIE* mutant became filamentous and seemed to contain several septa (Figure 5A and Supplementary Figures S2A,B). In *C. beijerinckii, spoIIE* disruption seems to impact the division site for asymmetric septation, as observed in *B. subtilis* (Barák and Youngman, 1996).

To localize the SpoIIE protein in the cells, SpoIIE was fused to the mCherry fluorescent protein (Ransom et al., 2015) and introduced into the Δ*spoIIE* strain. After 10 h of cultivation, atc was added in the cultures inducing the expression of the mCherry protein in both Δ*spoIIE* strains harboring, either pRAN73S or pRAN73S:cbei0097 plasmid. After induction, the cells were incubated for 8 h and then observed by fluorescent microscopy (Figure 5B and Supplementary Figure S2C). As described in *B. subtilis*, SpoIIE proteins seemed to gather mainly near the poles of the cell.

### Comparison of the Fermentation Profile of the Wild Type Strain and ΔspoIIE Mutant

**FIGURE 6:**
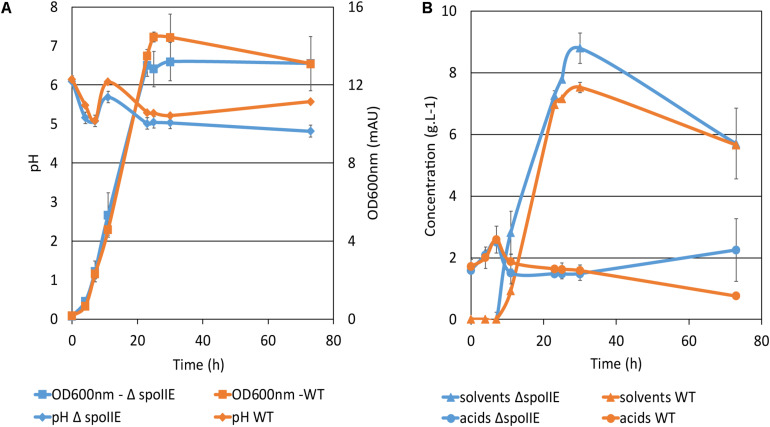
Fermentation profiles of *C. beijerinckii* strains. Fermentations were performed in biological and technical duplicates in CM2 medium in chemostats at 35°C during 73 h of cultivation. **(A)** Growth curve and pH variation in the cultures; **(B)** Acids and solvent titers during the fermentation. The error bars indicate one standard deviation of the mean, which was determined based on the data from biological and technical replicates (*n* = 4).

The WT and Δ*spoIIE* strains were grown in a chemostat. Growth, substrate consumption, product formation and pH were monitored for 73 h (Figure 6 and Table 2). The disruption of *spoIIE* did not affect biomass creation as the mutant strain reached the stationary phase after 23 h, like the WT strain (Figure 6A). As observed in small scale fermentations (Figure 3C), the mutant consumed glucose to produce acids and solvents. However, the pH in the Δ*spoIIE* mutant culture dropped slightly earlier than in the WT culture (Figure 6A). The reassimilation of the acids started earlier in the mutant culture as the solvent titer at 11 h was twice higher than in the mutant culture compared to the WT. After the reassimilation of butyrate and acetate, the pH at 11 h rose only to 5.7 ± 0.1 in the mutant culture while reaching 6.1 ± 0.0 in the WT culture. From 11 h to 23 h, the pH decreased in both cultures abruptly to 5.0 ± 0.1 in the Δ*spoIIE* mutant culture and 5.3 ± 0.0 in the WT culture. After 23 h, the pH increased slightly to 5.6 ± 0.0 in the WT culture but still decreased in the mutant culture to 4.9 ± 0.2. At the end of the fermentation, higher amounts of butyrate and acetate accumulated in the mutant culture. Indeed at 49 h, the acetate concentration reached 0.8 g.L^–1^ ± 0.2 in the mutant culture and 0.3 g.L^–1^ ± 0.0 in the WT culture. The concentration of acids after 73 h of cultivation in the chemostat was higher in the Δ*spoIIE* mutant culture, as observed in the serum bottles (Figure 3C). This difference is probably due to the absence of reassimilation of these acids in the Δ*spoIIE* mutant. The mutant also produced more acetone and butyric acid than the WT (Table 2); but, we did not observe an elongation of the solvent production phase but rather a switch to acidogenesis after 35 h.

**TABLE 2:** Fermentation data on substrate consumption and product formation of cultures of WT and Δ*spoIIE* strains after 30 h of cultivation in bioreactors, one standard deviation of the mean was determined based on the data from independent biological and technical duplicates (*n* = 4) * acetate was also produced by the mutant strain, n.d = not detected.

	Wild type	Δ*spoIIE*
**Substrates consumed [g.L^–1^]**		
Glucose	32.5 ± 0.5	37.0 ± 1.4
Acetate	1.1 ± 0.1	0.8 ± 0.1
**Products at the end [g.L^–1^]**		
Acetate*	0.5 ± 0.0	0.7 ± 0.1
Butyrate	0.6 ± 0.0	0.7 ± 0.1
Lactate	0.5 ± 0.1	0.1 ± 0.0
Acetone	1.9 ± 0.1	2.7 ± 0.2
Butanol	5.8 ± 0.1	6.3 ± 0.3
Ethanol	n.d	n.d

### Impact of the *spoIIE* Inactivation on the Transcriptome

#### Overview of the Transcription Data

**FIGURE 7:**
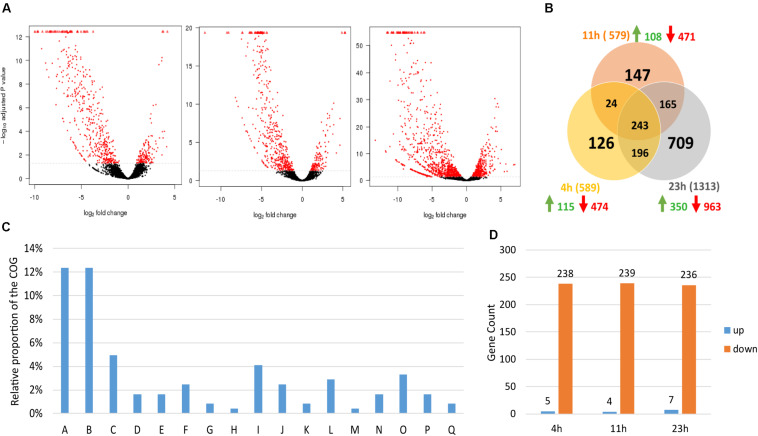
Overall differential expression dynamics. **(A)** Volcano plots of each comparison. Red dots represent significantly differentially expressed features; **(B)** Venn diagram showing the number of differentially expressed genes in the mutant at each time point; **(C)** COG class repartition of the 243 genes differentially expressed at all time points in the Δ*spoIIE* strain: A, Sporulation; B, Cell wall/membrane/envelope biogenesis; C, Amino acid transport and metabolism; D, Acidogenesis and Solventogenesis; E, Stress response; F, Other posttranslational modification, protein turnover, chaperones; G, Secondary metabolites biosynthesis, transport and catabolism; H, Coenzyme transport and metabolism; I, Carbohydrate transport and metabolism; J, Signal transduction mechanisms; K, Replication, recombination and repair; L, Inorganic ion transport and metabolism; M, Intracellular trafficking, secretion, and vesicular transport; N, Lipid transport and metabolism; O, Energy production and conversion; P, Transcription; Q, Defense mechanisms. **(D)** Proportion up and down-regulated genes at each time point in the cluster of the genes differentially expressed at all time points in the Δ*spoIIE* strain. The RNAseq data analyzed contains three independent biological replicates per time point and per strain (*n* = 3).

To study the repercussion of *spoIIE*’s disruption on the transcriptome, samples for mRNA isolation were collected from three independent chemostat fermentations of the WT and the *ΔspoIIE* mutant at three time points (4, 11, and 23 h), corresponding to early exponential, mid-exponential and entry into the stationary phase. After RNA isolation, library construction and sequencing, the data were mapped against the published genome (NCBI). The differential expression was calculated using the SARtools pipeline on the Institut Pasteur Galaxy platform (Varet et al., 2016). Out of the 5026 coding genes annotated by NCBI Prokaryotic Genome Annotation Pipeline (PGAP), 5021 were detected in our transcriptomic data. We then compared the expression profile of the WT and Δ*spoIIE* mutant strains. The inactivation of *spoIIE* had a significant impact on the transcriptome, as about 40% of the total CDS was differentially expressed in the mutant at least at one time point (2005 out of the 5021 genes transcribed). The differentially expressed genes were clustered using the Clusters of Orthologous Groups (COGs) database (NCBI). Thirty-seven percent of these 2005 genes encode for proteins of unknown function. The rest encodes mainly proteins involved in sporulation, metabolism, signal transduction, and the membrane/cell wall biogenesis. At each time point, more than 70% of the genes were significantly down-regulated (log_2_ fold change < −1.5 and padj < 0.05) (Figure 7A). The difference between mutant and WT transcriptome was the largest at 23 h. Indeed, while the number of differentially expressed genes with a | log_2_ fold | > 1.5 and padj < 0.05 was equal to 589 and 578 at 4 h and 11 h, respectively, it reached 1312 genes at 23 h. This increase at 23 h was mainly due to a rise in the number of differentially expressed genes involved in metabolism (from 147 genes at 11 h to 432 at 23 h) and encoding proteins of unknown function (from 218 to 469).

Two hundred and forty-three genes were significantly up or down-regulated (|log_2_ fold | > 1.5 and padj < 0.05) in the mutant compared to the WT at the three time points (Figure 7B and Supplementary Table S4), 97% of them being down-regulated at each time point in the Δ*spoIIE* mutant. And 45% of these 243 genes encode proteins of unknown function while the remaining genes encode proteins involved in various cellular processes such as sporulation, solventogenesis, or chemotaxis (Figure 7C). Only four genes were up-regulated at the three time points. They form an operon of 4 genes annotated as a histidine kinase, two diguanylate cyclases, and a transcription regulator.

To analyze in more detail the data, the genes were clustered according to the putative function of the encoded proteins. In addition to the differential expression of the genes involved in sporulation, the differential expression of the genes linked to five additional cellular processes was analyzed: stress response, cell wall and membrane formation, signal transduction and motility, carbohydrate metabolism, and amino acid, ion and vitamin transport and metabolism. The COG clustering of the differential expressed genes revealed that these five functional clusters were the most affected by SpoIIE’s inactivity after the sporulation cluster (Figure 7C).

#### Impact of the *spoIIE* Disruption on the Expression of Sporulation Genes

The genes involved in the sporulation process were the most down-regulated in the Δ*spoIIE* mutant. Indeed 84% of them are differentially expressed at least at one time point, and they have the lowest log_2_ fold change at 11 h and 23 h (Supplementary Table S5). To better assess the impact of *spoIIE*’s disruption, the genes involved in sporulation regulation were clustered based on the genes present in *C. beijerinckii*’s genome and on previous studies on sporulation in *C. acetobutylicum* (Al-Hinai et al., 2015), *C. difficile* (Saujet et al., 2013; Fimlaid et al., 2013) and *B. subtilis* (Wang et al., 2006; Zhu and Stülke, 2018). According to the data concerning the corresponding regulon in *C. acetobutylicum*, in *C. difficile* and *B.subtilis*, the genes involved in sporulation were divided into six groups: initiation of sporulation, Spo0A regulon, σ^*F*^ regulon, σ^*E*^ regulon, σ^*G*^ regulon and σ^*K*^ regulon (Supplementary Table S6). However, 23 genes, homologs to known sporulation genes, could not be linked to any known regulon.

The expression levels of most genes involved in the initiation of the sporulation and the activation of Spo0A by phosphorylation did not change in the Δ*spoIIE* mutant (Supplementary Table S6). The expression of *spo0A* remains rather stable. Only the expression of two genes (Cbei_4885 and Cbei_3375) encoding AbrB-type regulators significantly differed in the mutant compared to the WT. In *C. acetobutylicum*, *abrB* genes were shown to have a crucial role in the transition from acidogenesis to solventogenesis (Xue et al., 2016). Out of the three *abrB* genes identified in *C. acetobutylicum*, two (Cac_3647 and Cac_0310) encode transcriptional regulators down-regulating genes necessary for solventogenesis: the *sol* operon as well as *ald, bdh, adhE2* and *adc* genes. In *C. beijerinckii*, five *abrB* genes, including Cbei_3375 and Cbei_4885, are annotated. Cbei_4885 is highly similar to Cac_3647 and Cac_0310 with 88 and 89% identity, respectively. The expression of Cbei_4885 increased at 11 h and decreased at 23 h (log_2_ fold change of 2.09 and −2.75) while the expression of Cbei_3375, 54% identity with Cac_3647 and Cac_0310, increases slightly at 23 h (log_2_ fold change of 1.86).

In the Δ*spoIIE* strain, expression levels of the sporulation genes belonging to the Spo0A regulon were similar to those of the WT strain. As expected, no mRNA of the *spoIIE* gene was detected in the mutant cells. The expression level of *spoIIGA* and *sigE* was not differentially expressed in the mutant, at 4 h log_2_ fold change is low (−2.69 and −2.60) but was not statistically relevant, padj > 0.05. At 11 h and 23 h, their expression returned to WT levels. The *spoIIAA-spoIIAB-sigF* operon was also slightly less expressed at 4 h in the mutant compared to the WT, as observed for the *spoIIGA-sigE* operon. As seen in other spore formers (Fujita et al., 2005; Saujet et al., 2014), Spo0A boxes were detected upstream from these operons, suggesting that Spo0A controls their expression.

By contrast, the expression of genes involved in the later stages of the sporulation was more severely impacted by *spoIIE*’s disruption. The genes belonging to the σ^*F*^ regulon were all down-regulated at the three time points except *spoIIR*, suggesting that *spoIIR* is not part of the σ^*F*^ regulon in *C. beijerinckii*, in contrary to its homolog in *B. subtilis*. Also, the fold-change of expression varied throughout the σ^*F*^ regulon. Indeed, while some genes like *spoIIQ*, *spoIIP* and *spoIVB* were strongly down-regulated at the three time points (Supplementary Table S6), other genes like *gpr* and *dacF* were mainly down-regulated at 4 h and 23 h. In the case of *dacF*, this could be due to the low coverage of the gene already in the WT at 11 h, but that was not the case for *gprR*. This result suggests that another transcription factor might be involved, enabling the expression of these genes during exponential growth in the *spoIIE* mutant. Most genes involved later in the sporulation process (Stage III to VII) belonging to the σ^*E*^, σ^*G*^, or σ^*K*^ regulon, were down-regulated. Only four genes (*spoVD1*, *spoVD2*, *spoVE* and *yzbD*), associated with the σ^*E*^ regulon in *B. subtilis*, were not differentially expressed in our strain.

However, out of the 50 most differentially expressed genes at 11 h and 23 h (Supplementary Table S5), only 14% and 24% of genes correspond to known proteins directly involved in the sporulation process, while 54% and 58% of the genes, respectively were of unknown function. This large number of genes with unknown function shows that other genes might be involved in the sporulation process and that *spoIIE* inactivation and the cells block at stage II of the sporulation cycle has an impact on other gene clusters.

#### The Disruption of *spoIIE* Affects Other Cellular Processes

As observed in the COG clustering of the genes three times differentially expressed, the inactivation of *spoIIE* has an impact on several cellular processes besides sporulation, including the central metabolism (Figure 7C). Indeed the results that were obtained in this study showed the differential expression of genes associated with five functional groups: stress response, cell wall and membrane formation, signal transduction mechanisms and motility, carbohydrate metabolism, and amino acid, ions, and vitamin transport and metabolism.

##### Stress response

Genes involved in stress response were also strongly differentially expressed in the Δ*spoIIE* mutant (Supplementary Table S7). The genes coding for rubrerythrins (Cbei_0569, Cbei_2325, and Cbei_3257) and superoxide dismutase (Cbei_1507, Cbei_1856) belonging to the σ^*G*^ regulon in *C. difficile* (Saujet et al., 2013) were down-regulated in the mutant at all time points. In contrast, the genes coding for chaperonin proteins Cpn10, GroEL, and DnaJ, known to be involved in butanol tolerance in *C. acetobutylicum* (Tomas et al., 2004), were up-regulated at 23 h.

##### Cell wall and membrane formation

As shown in the microscopy pictures (Figures 4, 5 and Supplementary Figures S2A,B), the morphology of Δ*spoIIE* mutant cells is very different from WT cells’. Thus, a difference in the expression of genes encoding membrane- and cell wall-associated proteins (Supplementary Table S7) was expected. Among the 243 genes differentially expressed in the mutant at the three time points, 42 belonged to the cell wall/membrane cluster. These 42 genes were all down-regulated in the mutant (log_2_ fold change ranging from −12 to −1.9). Several of them encode glycosyltransferases, which might suggest a possible modification of the cell wall. An up-regulation of the genes involved in septum formation, *fstZ, minD* and *refZ* was also observed. These genes were proven to be crucial for the positioning of the septum formation and sporulation in *B. subtilis* (Barák and Muchová, 2018; Brown et al., 2019). This result can be linked to the presence of several septa observed in the mutant (Figure 5 and Supplementary Figures 2A,B).

##### Signal transduction mechanisms and motility

The initiation of the sporulation cycle is triggered by several environmental signals (Al-Hinai et al., 2015). Environmental changes are detected and processed by the cells through several transduction mechanisms. In *C. acetobutylicum*, two quorum sensing mechanisms were studied, an Agr system (Steiner et al., 2012) and an RNPP type system (Kotte et al., 2017). Homologs of the Agr system were identified in *C. beijerinckii*, but no homologs of the RNPP proteins were found. In the Δ*spoIIE* mutant, the *agr* operon was not differentially expressed. In *C. acetobutylicum*, another type of bioactive metabolite, the clostrienose, synthesized by a PKS system, is also involved in the control of solvent production and sporulation, mainly through σ^*K*^ activation (Herman et al., 2017). No homolog to the clostrienose producing PKS enzyme was found in *C. beijerinckii*. Still, a PKS-NRPS gene cluster of 64.5 kb (Letzel et al., 2013) is present, enabling the secretion of a secondary metabolite, circularin A (Kemperman et al., 2003). This cluster of 50 genes, from Cbei_0233 to Cbei_0283, encodes biosynthetic proteins (Cbei_0249-0254), and four transporters (Cbei_0260-0264). These genes were differentially expressed in the mutant (Supplementary Table S8). The genes encoding the biosynthetic proteins are up-regulated at 4 h (log_2_ fold change from 1.9 to 2.9) and down-regulated at 23 h (log_2_ fold change from −4.6 to −1.1) while the genes encoding transporters were up-regulated at 4 h and down-regulated at 11 h.

About 20% of the genes involved in signal transduction are differentially expressed at least one time point in the Δ*spoIIE* mutant. Several genes encoding serine/threonine kinases and PAS/PAC sensor hybrid histidine kinases were differentially expressed. In particular, six genes were differentially expressed at all three time points. Genes encoding two serine/threonine kinases and an unknown gene were consistently down-regulated (log_2_ fold change between −10 and −3.8). Three genes belonging to the same operon were up-regulated (log_2_ fold change between 2.7 and 4.2). This operon is unique to *C. beijerinckii.* It harbors a PAS/PAC sensor hybrid histidine kinase, a diguanylate cyclase/phosphodiesterase with PAS/PAC sensor(s) and a diguanylate cyclase with a transcription regulator.

In *Clostridium*, the loss of motility is usually coupled with the transition from exponential phase to stationary phase (Gutierrez and Maddox, 1987; Wang et al., 2012). At 4 h, no significant differential expression was observed for chemotaxis and motility genes (Supplementary Table S7) in the mutant. However, at 11h and 23 h, respectively, 33% and 16% of genes from this cluster were up-regulated in the *spoIIE* mutant. The chemotaxis gene operon Cbei_4307 to Cbei_4312, as well as the motility genes *motA* and *motB*, were more transcribed in the mutant compared to the wild type with a log_2_ fold change between 2 and 5 (Supplementary Table S7).

##### Carbohydrate metabolism

###### Granulose biosynthesis

**FIGURE 8:**
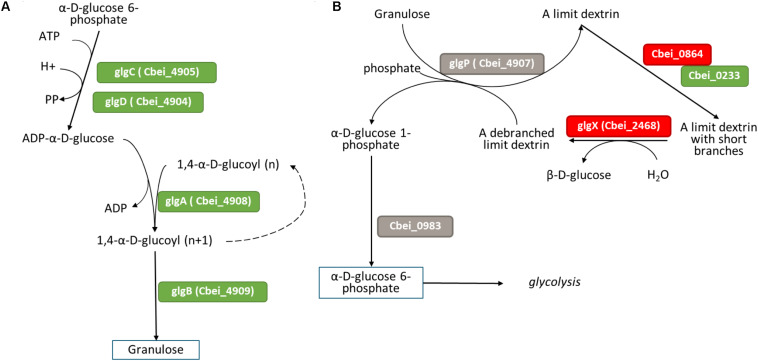
Differential expression at 23 h in the mutant of genes involved in **(A)** granulose formation and **(B)** granulose degradation figures adapted from Microcyc. Gene names are in colored boxes according to the differential expression in the Δ*spoIIE* mutant compared to the WT; genes in gray boxes have no change in expression, genes in green boxes are up-regulated, and genes in red boxes are down-regulated.

Granulose, also called bacterial glycogen, is a starch-like polymer produced at the onset of the sporulation cycle (Robson et al., 1974). Granulose biosynthesis is one of the indicators of sporulation initiation. No differential expression of granulose biosynthesis genes was observed at 4 h or 11 h (Supplementary Table S9). However, at 23 h (Figure 8A), the genes *glgA, glgC* and *glgD* involved in granulose formation were all up-regulated in the Δ*spoIIE* mutant strain. In contrast, the expression of genes involved in granulose degradation (Cbei_0983 and *glgP*) remained unchanged (Figure 8B). The regulation of granulose synthesis by the Agr quorum-sensing system has been described in *C. acetobutylicum* (Steiner et al., 2012). However, no differential expression of the agr operon was observed at any time point in our dataset. As described in *E. coli* (Eydallin et al., 2007; Montero et al., 2009; Wilson et al., 2010), for the molecular regulation of glycogen formation, granulose regulation might be more complex, involving several transcriptional regulators such as CsrA and PhoP/PhoQ. While no difference in *csrA* expression was detected, both *phoP* and *phoQ* genes were up-regulated at 23 h (log_2_fold change of 2.812 and 2.21). In *E. coli*, PhoP and PhoQ promote the expression of the glycogen formation genes *glgA, glgC* and *glgD*, which could be the case for granulose formation in the *spoIIE* mutant.

###### Central metabolism

**FIGURE 9:**
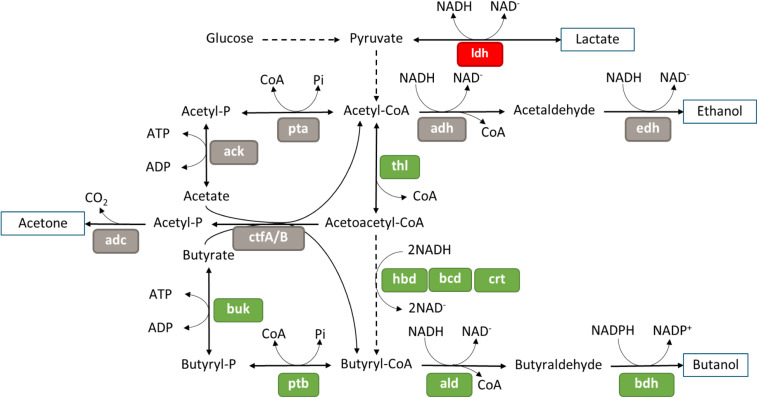
Differential expression in the mutant at 23 h of genes involved in acids and solvent formation, figure adapted from Microcyc. Gene names are in colored boxes according to the differential expression in the Δ*spoIIE* mutant compared to the WT; genes in gray boxes have no change in expression, genes in green boxes are up-regulated and genes in red are down-regulated.

Most of the genes involved in glycolysis were not differentially expressed in the Δ*spoIIE* mutant (Supplementary Table S9). Only one of the two copies of the pyruvate kinase, Cbei_1412, was down-regulated at the three time points, but no change in expression was observed for the second copy Cbei_4851. However, half of the genes involved in acidogenesis and solventogenesis were differentially expressed in the mutant compared to the WT strain at 23 h (Figure 9). Indeed, while the genes putatively involved in lactate and ethanol production were down-regulated in the mutant strain, the genes involved in butyrate and butanol production were up-regulated. However, no change in the expression of the genes coding for the proteins involved in acetate or acetone production was detected. The up-regulation of the *sol* operon, as well as genes encoding several alcohol dehydrogenases, might be linked to the down-regulation of the *abrB* gene, Cbei_4885, as a similar control was observed in *C. acetobutylicum* when its homolog was disrupted. We saw no differential expression of the *ctfA/B* operon, which could be linked to the accumulation of butyrate observed in the fermentation broth after 23 h, despite the up-regulation of the other genes involved in the butanol production.

##### Amino acid, ions and vitamin transport and metabolism

Several vitamins and ions are essential for solventogenesis and sporulation in *Clostridium* (Jamroskovic et al., 2016; Nimbalkar et al., 2018; Li et al., 2018; List et al., 2019). These findings contributed to the modification of growth media to improve either the sporulation or the solvent formation. As shown in a previous transcriptomic study on *C. beijerinckii* (Vasylkivska et al., 2019), the expression of genes involved in amino acids, vitamins, and ions transport varied depending on the culture’s growth stage. Based on this work, close to 600 genes, putatively involved in amino acid, ions and vitamin transport and metabolism, were identified. The transcription of 32% of them differed in the Δ*spoIIE* mutant at one time point, at least (Supplementary Table S10). Twenty-one genes, including the *app* operon, were down-regulated in the mutant at the three time points. In *B. subtilis* and *C. difficile*, the *app* operon codes for permeases allowing the uptake of small quorum signaling peptides essential for the initiation of sporulation (Edwards et al., 2014).

At 4 h genes involved in ascorbate, cobalt and iron transport were down-regulated while genes encoding riboflavin and vitamin D transporters were upregulated. Moreover, at 11 h and 23 h, genes coding for cysteine-, glutamine- and manganese-transporters and the metabolism of these compounds were strongly down-regulated in the mutant (log_2_ fold change between −11 and −4). By contrast, we observed an up-regulation of genes encoding a proline transporter (Cbei_2870 and Cbei_2871) that has been linked to a rise in proline in the cell, which may act as a stress protectant (Takagi, 2008; Lee et al., 2015). At the same time points, the genes coding for ferritin and the niacin transporter were up-regulated. Niacin is a precursor of NAD(H) and NADP(H), and its presence in the medium can lead to an increase in butanol production (Li et al., 2014). The up-regulation of the niacin transporter could be explained by a lack of NAD(H) in the cell, which can be linked to the observed accumulation of butyrate.

At 23 h, the genes linked to the transport of phosphate were up-regulated. By contrast, the genes coding for methionine and glycine/betaine transporters (log_2_ fold change from −5 to −4) were strongly down-regulated as well as the genes encoding for ascorbate- and iron transporters. The up-regulation of ferritin, coupled with the down-regulation of iron transporters, indicates an accumulation of iron in the cell, requiring the action of ferritin and an interruption of the iron uptake. The role of iron in sporulation in *Clostridium* is not clear. In *C. botulinum*, iron is needed for the formation of heat resistant spores while in *C. sporogenes*, its addition impairs sporulation (Mah et al., 2008). In the case of *C. beijerinckii*, the data suggests that iron is mostly needed for sporulation. The Δ*spoIIE* mutant limits its uptake and has to store the excess of iron present in the cell.

##### Genes of unknown function

Our transcriptomic analysis highlighted the presence of a large cluster of genes of unknown function impacted by *spoIIE*’s inactivation. Indeed, 45% of the genes differentially expressed at the three times points did not have a known function. Except for one gene coding for a histidine triad (HIT) protein up-regulated at 23 h, these genes were down-regulated at all time points. Moreover, when focusing only on the genes differentially expressed at the 23 h, 56% of the most differentially expressed genes (log_2_ fold change above 5) belonged to the unknown function cluster. Some of these strongly down-regulated genes might code for spore coat proteins. Indeed, some coat proteins are species-specific and of small size, and they remain to be identified in *C. beijerinckii* (de Hoon et al., 2010). Spore coat proteins mainly belong to σ^*E*^ and σ^*K*^ regulon in *B. subtilis* and *C. difficile*. In *C. difficile*, the expression of a few genes encoding spore coat proteins is regulated by σ^*G*^ (Paredes-Sabja et al., 2014). Spore coat genes are expressed at stages IV-VII of sporulation (Al-Hinai et al., 2015). The Pan/Core Genome analysis tool (Miele et al., 2011) was used to investigate whether some differentially expressed genes of unknown function were species-specific. Thirteen genomes of 5 clostridial species (*C. acetobutylicum, C. beijerinckii, C. butyricum, C. saccharolyticum, C. saccharoperbutylicum*) were compared. One hundred and seventy-two genes of unknown function were down-regulated at 23 h were species-specific. Amongst them, thirty-one genes encoded small proteins, shorter than 100 amino acids that were strongly down-regulated at 23 h (Supplementary Table S11).

## Discussion

*Clostridium beijerinckii* is a solventogenic bacterium that is well-studied because of its potential to produce biofuels and biochemicals (Lépiz-Aguilar et al., 2011; Maiti et al., 2016). However, while its central metabolism has been studied in detail, only a few studies on the regulation of its sporulation were conducted up to date. As *C. beijerinckii* belongs to the solventogenic clostridia, it has been assumed that its sporulation mechanism is identical to the one described in *C. acetobutylicum*. In this study, we described the phenotype of a Δ*spoIIE* mutant in *C. beijerinckii*, generated by CRISPR-Cas9, to verify this assumption. CRISPR-Cas9 genome engineering enables the generation of a more stable phenotype than the methods used for the *C. acetobutylicum spoIIE* mutants, generated by RNA silencing (Scotcher and Bennett, 2005) and single-crossover (Bi et al., 2011). As described in *C. acetobutylicum*, the Δ*spoIIE* mutant of *C. beijerinckii* was not able to produce viable spores. The mutant was also able to produce solvents and acids at a higher level than the WT (Bi et al., 2011). Moreover, we confirmed the presence of granulose in the mutant cells, while granulose production was not tested in the *C. acetobutylicum*Δ*spoIIE* mutant. Granulose formation is the first visible indication of the cell’s entry in the sporulation cycle. However, the regulatory network linking granulose formation and sporulation has not been elucidated. While several studies report that granulose and solvent formation can be totally decoupled (Reysenbach et al., 1986; Tracy et al., 2011), this is not the case for sporulation and granulose formation (Steiner et al., 2012). This study describes the first solventogenic asporogenous *Clostridium* mutant able to produce granulose.

**FIGURE 10:**
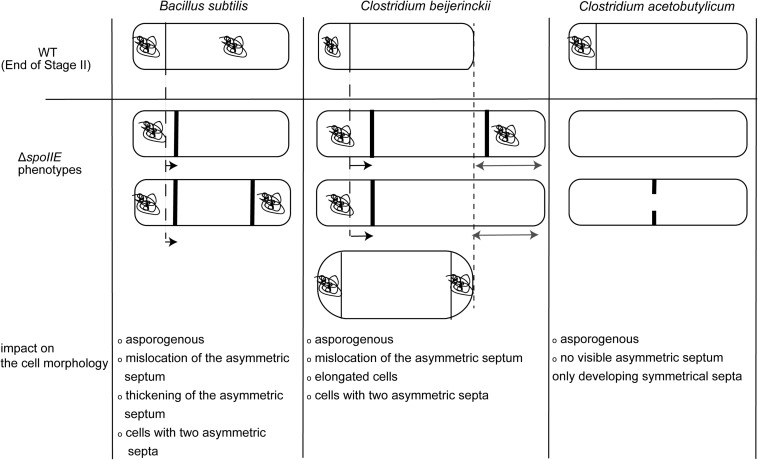
Schematic representation of the differences in cell morphology of Δ*spoIIE* mutants in *B. subtilis*, *C. beijerinckii* and *C. acetobutylicum*. The figure was constructed from data reported in this study and in Barák and Youngman (1996), Al-Hinai et al. (2015), Barák and Muchová (2018).

Furthermore, the morphology of Δ*spoIIE* mutants in both clostridia is also different. In contrast to *C. acetobutylicum* Δ*spoIIE* cells, *C. beijerinckii* Δ*spoIIE* cells were elongated with several septa and showed phase-dark bodies at their polar ends. *C. acetobutylicum* mutant cells do not display any differentiation phenotype. Indeed *C. acetobutylicum*Δ*spoIIE* cells looked like vegetative cells; no internal structures apart from symmetrical septal membranes or other changes in morphology were described, even after 72 h of cultivation. However, in *B. subtilis*, the described Δ*spoIIE* mutant has a morphology close to the one observed in our study (Barák and Youngman, 1996), as illustrated in Figure 10. We can thus hypothesize that the role of SpoIIE in *C. beijerinckii* more closely resembles the function described in *B. subtilis* (Al-Hinai et al., 2015). In *B. subtilis*, SpoIIE enables the activation of σ^*F*^ and the positioning of the asymmetric septum, a second role that has not been described for *C. acetobutylicum*.

To better understand the role of SpoIIE, we compared the expression profile between the *spoIIE* mutant and WT strains by RNA sequencing at early-, mid-exponential and stationary phases. Forty percent of the total CDS were differentially expressed between both strains, with the 23 h time point showing the most changes. The genes involved in the regulation of sporulation after stage II were down-regulated after 23 h of culture. In contrast to what was observed in *C. acetobutylicum*, *sigF, sigE* and *sigG* were still expressed at 11 h. In *C. acetobutylicum*, a significant decrease of *sigF* transcript and σ^*F*^ production in the Δ*spoIIE* mutant was observed (Bi et al., 2011), suggesting that the impact of *spoIIE* inactivation on *sigF* expression is less pronounced in *C. beijerinckii*. The steady expression of *sigF* at 11 h in the *C. beijerinckii* Δ*spoIIE* mutant also suggests that σ^*F*^ does not influence its own expression, which contrasts with the regulation model described in *C. acetobutylicum* (Al-Hinai et al., 2015). In addition, the inactivation of *spoIIE* or *sigF* abolishes *sigE* expression and σ^*E*^ production in *C. acetobutylicum* (Bi et al., 2011 and Jones et al., 2011) while this is not the case for *sigE* expression in *C. beijerinckii*.

The *sigE* and *sigG* transcripts were slightly less abundant in the mutant than in the WT. These results suggest that in *C. beijerinckii*, the expression of *sigE* and *sigG* is only partially regulated by σ^*F*^. Despite being expressed, σ^*E*^ and σ^*G*^ might not be functional since the genes belonging to their respective regulons were weakly expressed in the mutant. This observation could be explained by (i) the need for σ^*E*^ and σ^*G*^ to reach a certain threshold level in the cell for them to allow the transcription of their regulons, (ii) a lack of active σ^*E*^ in the mother cell. Pro-σ^*E*^ might not be processed in the mutant and σ^*E*^ might then stay inactive. In *B. subtilis*, the SpoIIGA protease processes Pro-σ^*E*^, after being activated by SpoIIR, which is expressed under the control of σ^*F*^. It is interesting to note that in *C. beijerinckii*, we failed to detect a clear impact of *spoIIE* inactivation on s*poIIGA* and *spoIIR* expression, even though *spoIIR* belongs to the σ^*F*^ regulon in *B. subtilis*. Thus, another protein, belonging to the σ^*F*^ regulon might be required to process Pro-σ^*E*^. It is worth noting that also in other clostridia, the expression of *spoIIR* is not strictly dependent on σ^*F*^ (Jones et al., 2011; Saujet et al., 2013).

The transcriptomic data confirmed that next to sporulation, other stationary-phase phenomena such as stress response, signal transduction, motility, carbohydrate metabolism, and the transport of amino acids, vitamins and ions were modified in the Δ*spoIIE* mutant. The interruption of the sporulation cycle led to an extension of the metabolic activity of the cells. Thus, the asporogenous Δ*spoIIE* mutant displayed three exciting features that, as observed in other solventogenic clostridia, appear to be associated with an increase in solvent production. Firstly, several genes involved in butyrate and butanol production were up-regulated. While a definite rise in acid titer was observed in the medium at the end of the fermentation, no substantial increase in butanol was measured. This rise in acid titer might have been caused by a lack of CtfA/B proteins. The CtfA/B complex enables the reassimilation of acids; however, their genes were not up-regulated in the Δ*spoIIE* mutant. The introduction of these genes in the Δ*spoIIE* mutant, expressed under the control of a constitutive promotor, might enhance the solvent production, as previously observed in *C. acetobutylicum* (Collas et al., 2012). Another explanation for the increase of the acid titer could be the lack of reducing power to convert the CoA esters into alcohols. Secondly, the genes encoding the chaperone proteins GroESL and DnaJ were up-regulated. Overexpression of these heat shock proteins in *C. acetobutylicum* increases tolerance to chemical stress (Tomas et al., 2003; Liao et al., 2017). Lastly, a change in the expression of genes encoding transporters of amino acids, ions and vitamins was observed in the Δ*spoIIE* mutant. These results suggest that a modification of the medium could also increase the solvent titer. Indeed, the addition of niacin has been demonstrated to improve butanol production (Li et al., 2014).

In conclusion, this study has enabled us to identify genes potentially involved in the regulation of stationary phase phenomena. The expression of genes involved in secondary metabolism and signaling pathways, as well as a large number of genes of unknown functions, were strongly impacted in the *spoIIE* mutant. These genes should be investigated in more detail to obtain a better insight into the regulation of sporulation and other stationary-phase events. Indeed, the complete sporulation regulation pathway has not yet been elucidated; critical information such as activation of σ^*G*^ (Al-Hinai et al., 2015) or the spore coat composition of *C. beijerinckii* are unknown.

The impact of the interruption of the sporulation cycle at Stage II in *C. beijerinckii* was evaluated through fermentation, microscopy, genome and transcriptome analysis. The fermentation potential of the mutant strain could be evaluated. The transcriptomic analysis provided directions to better exploit this potential, by gene engineering or specific changes in media composition. Furthermore, this study reveals the complexity of the regulation of sporulation in *Clostridium* and its interconnection with other cellular regulatory networks. While some features are conserved, others seem to differ even between solventogenic clostridia. Nevertheless, this work constitutes a solid basis for further investigation of the molecular regulation of sporulation in *C. beijerinckii* and its potential for industrial application.

## Data Availability Statement

The RNA seq data generated and analyzed for this study have been deposited in the ArrayExpress database at EMBL-EBI (www.ebi.ac.uk/arrayexpress) under accession number E-MTAB-7481. The genome sequence of *Clostridium beijerinckii* NCIMB 8052 Δ*spoIIE* strain described in this study is available on the European Nucleotide Archive (ENA) under the accession number PRJEB39199.

## Author Contributions

MD co-designed the study, performed the experiments, collected and analyzed the data, and wrote the manuscript. NK collected the transcriptomic data. MM contributed to the transcriptomic data analysis. FC supervised the experimental work. IM-V, JO and SK contributed to data interpretation, discussions and revised the manuscript. AMLC co-designed the study, supervised the work, contributed to data interpretation, discussions and revised the manuscript. All authors contributed to the article and approved the submitted version.

## Conflict of Interest

The authors declare that the research was conducted in the absence of any commercial or financial relationships that could be construed as a potential conflict of interest.

## Footnotes

1 http://mmonot.eu/COV2HTML/

## Supplementary Material

FIGURE S1 Complementation of the Δ*spoIIE* mutant restores sporulation **(A)** Phase-contrast microscopy pictures (x400) after 35 h of cultivation of the Δ*spoIIE* mutant and the complemented strain; **(B)** Growth on plates after heat-shock treatment of 48 h old Δ*spoIIE* and complemented Δ*spoIIE* cultures. The short dark blue arrows indicate mature spores.

FIGURE S2 Additional fluorescence microscopy images of wild type and Δ*spoIIE* mutant cells; **(A)** Images of several WT and Δ*spoIIE* mutant cells after 20 h of cultivation and stained by the membrane staining MTG; **(B)** Image of two Δ*spoIIE* mutant cells after 20 h of cultivation and stained by the membrane staining MTG; **(C)** Fluorescence images of the Δ*spoIIE* mutant cells stained by MTG and harboring either the mCherry empty plasmid (pRAN73S) or the plasmid expressing mCherry fused to Cbei_0097 (pRAN73S:cbei_0097) at 18 h of cultivation (after 8 hours of atc induction). The white arrows indicate septa observed in the mutant strain.

TABLE S1 Oligonucleotides used in this study; the restriction sites are underlined.

TABLE S2 Sequences of primers used in Q-RT-PCR experiments for the validation of the RNAseq analysis and Q-RT-PCR values.

TABLE S3 SNPs detected in each Δ*spoIIE* mutant.

TABLE S4 Genes significantly differentially expressed in the Δ*spoIIE* mutant at the three time points and their expression levels in the mutant relative to the wild type in log_2_ fold change.

TABLE S5 Most differentially expressed genes at 11 h and 23 h and their expression levels in the mutant relative to the wild type in log_2_ fold change.

TABLE S6 Differential expression of the genes belonging to the sporulation cluster in log_2_ fold change; the genes in italic were not significantly differentially expressed (| log_2_ fold| < 1.5 or padj > 0.05) during the fermentation.

TABLE S7 Differential expression of the genes involved in stress response, cell wall/membrane composition, chemotaxis and motility in the Δ*spoIIE* mutant relative to the wild type, in log_2_ fold change; only the genes for which | log_2_ fold| > 1.5 and padj ≤ 0.05 at least once during the fermentation.

TABLE S8 Differential expression of the PKS-NRPS gene cluster in the Δ*spoIIE* mutant relative to the wild type, in log_2_ fold change; the genes in italic were not significantly differentially expressed (| log_2_ fold| < 1.5 or padj > 0.05) during the fermentation.

TABLE S9 Differential expression of the genes involved in the granulose formation, glycolysis, acidogenesis and solventogenesis in the *spoIIE* mutant relative to the wild type, in log_2_ fold change; the genes in italic were not significantly differentially expressed (| log_2_ fold| < 1.5 or padj > 0.05) during the fermentation.

TABLE S10 Differential expression of the genes involved in the amino acid, ion and vitamin transport and metabolism in the *spoIIE* mutant relative to the wild type, in log_2_ fold change; only the genes for which | log_2_ fold| > 1.5 and padj ≤ 0.05 at least once during the fermentation.

TABLE S11 Differential expression at 23 h of small species-specific genes of unknown function down-regulated in the Δ*spoIIE* mutant relative to the wild type, in log_2_ fold change; genes in bold are down-regulated at all time points.

TABLE S12 Fermentations characteristics of WT, Δ*spoIIE* and Δ*spoIIE* pSpoIIE complemented mutant after 70 h of cultivation in CM2 media in serum bottles at 35°C, one standard deviation of the mean was determined based on the data from biological replicates (*n* = 2).
