# Supplementary figures and images for: Transcriptomic and Phenotypic Analysis of a spoIIE Mutant in Clostridium beijerinckii
Source: Front Microbiol. 2020 Sep 15;11:556064. doi: 10.3389/fmicb.2020.556064 (PMC7522474; doi:10.3389/fmicb.2020.556064)

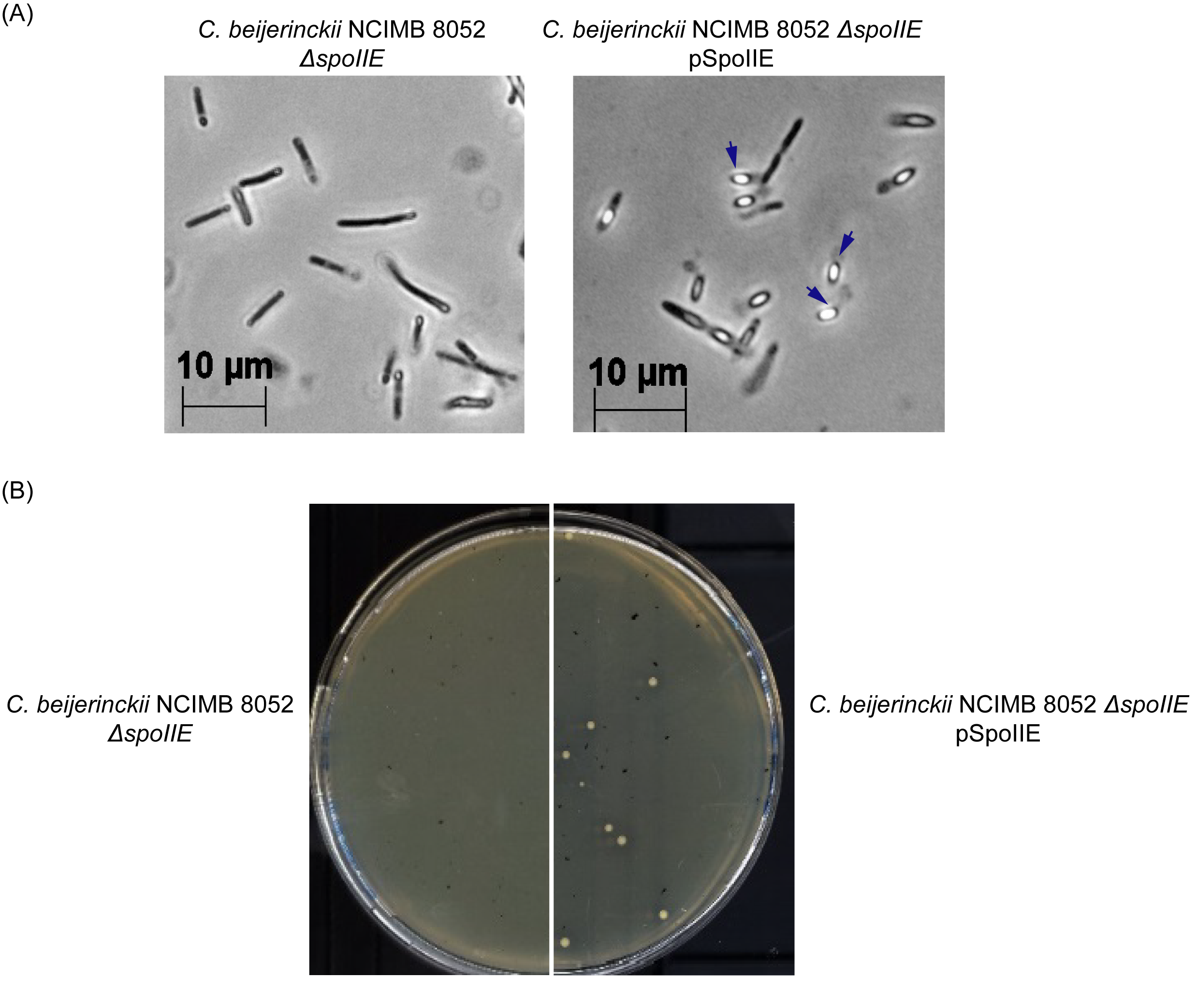

Supplement: FIGURE S1 — Complementation of the ΔspoIIE mutant restores sporulation (A) Phase-contrast microscopy pictures (x400) after 35 h of cultivation of the ΔspoIIE mutant and the complemented strain; (B) Growth on plates after heat-shock treatment of 48 h old ΔspoIIE and complemented ΔspoIIE cultures. The short dark blue arrows indicate mature spores. [file Image_1.TIF]

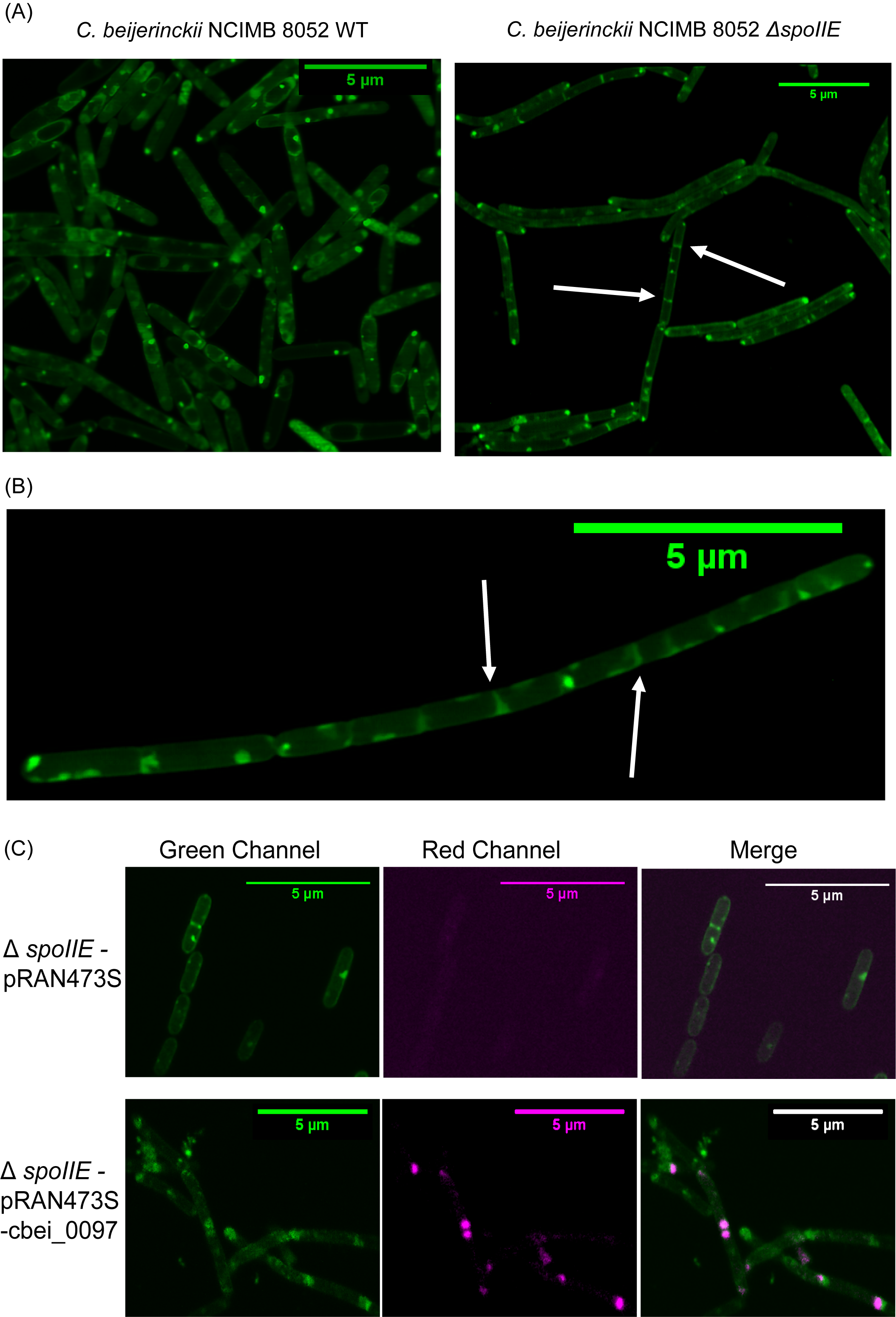

Supplement: FIGURE S2 — Additional fluorescence microscopy images of wild type and ΔspoIIE mutant cells; (A) Images of several WT and ΔspoIIE mutant cells after 20 h of cultivation and stained by the membrane staining MTG; (B) Image of two ΔspoIIE mutant cells after 20 h of cultivation and stained by the membrane staining MTG; (C) Fluorescence images of the ΔspoIIE mutant cells stained by MTG and harboring either the mCherry empty plasmid (pRAN73S) or the plasmid expressing mCherry fused to Cbei_0097 (pRAN73S:cbei_0097) at 18 h of cultivation (after 8 hours of atc induction). The white arrows indicate septa observed in the mutant strain. [file Image_2.TIF]
